# Carpal Tunnel Syndrome

**Published:** 2013-01-18

**Authors:** Milind Kachare, Edward Hahn, Mark S. Granick

**Affiliations:** Division of Plastic Surgery, New Jersey Medical School, University of Medicine and Dentistry, Newark, NJ

**Figure:**
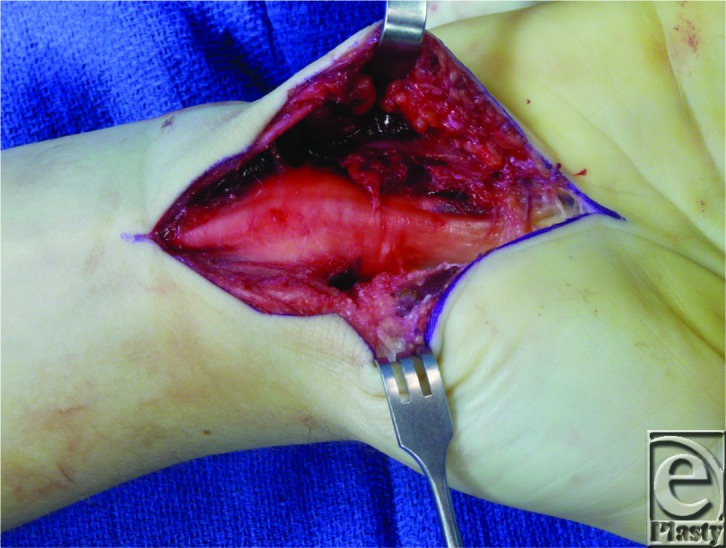


## DESCRIPTION

This patient had chronic medial nerve compression at the carpal tunnel. After release, the nerve appears thinned at the entrance to the carpal tunnel and bulging just proximal to the carpal tunnel.

## QUESTIONS

1. **What is the etiology of carpal tunnel syndrome (CTS)?**
2. **What anatomic structures are present in the carpal tunnel?**
3. **What are the surgical and nonsurgical treatment options for CTS?**
4. **What are the major complications of carpal tunnel release surgery?**
5. **Should the ulnar nerve be released as well?**

## DISCUSSION

Carpal tunnel syndrome is a common upper extremity disorder, and the most common mononeuropathy.^1^ The main cause of CTS is increased pressure within the carpal tunnel, which consequently applies compressive force to the median nerve. The increased pressure upon the median nerve causes nerve ischemia, which is the source of the motor and sensory dysfunction experienced by patients inflicted with CTS. Most cases are idiopathic but are associated with other conditions such as obesity, hypothyroidism, arthritis, diabetes, and trauma.

The carpal tunnel contains the median nerve and 9 tendons: the flexor digitorum superficialis and profundus tendons, as well as the flexor pollicis longus. The transverse carpal ligament forms the volar aspect of the carpal tunnel. The carpal bones are responsible for creating the lateral and dorsal boundaries of the carpal tunnel.

Increased pressure on the median nerve can affect the structures innervated by the median nerve distal to the wrist. The median nerve branches to give off a recurrent branch, which provides motor innervation to the thenar muscles: the abductor pollicis brevis, the flexor pollicis brevis, and the opponens pollicis. It also gives off digital cutaneous branches, which supply sensory innervation to the first 3 digits and half of the fourth digit on the palmar side. In addition, the median nerve supplies motor innervation to the first and second lumbricals of the hand.

Carpal tunnel syndrome remains a clinical diagnosis because there is no criterion standard test for the condition.^2^ Certain intrinsic predisposing factors of CTS are female gender, pregnancy, diabetes, and rheumatoid arthritis. A clinical diagnosis of CTS consists of the patient's history and a physical examination. Typical symptoms are pain and paresthesias of the palmar radial hand, which is worse at night and often awakens the patient from sleep.^1^ Physical examination may reveal thenar wasting, intrinsic muscle weakness, and impaired sensation in the distribution of the median nerve. The Phalen test and the Hoffmann-Tinel sign assist in deducing the diagnosis; they possess a specificity that ranges from 54% to 98% and from 55% to 100% and a sensitivity of 42% to 85% and 38% to 100%, respectively.^3^ Factors believed to contribute to CTS include repetitive or forceful tasks, and mechanical stress of the hands.^4^ Physicians often employ electrodiagnostic tests to assist in deducing the clinical diagnosis and locating areas of compressive neuropathy. Still, the physical examination is the hallmark tool in making a diagnosis of CTS.^2^

Acute compression of the median nerve within the carpal tunnel can occur with trauma, such as a wrist fracture or dislocation. This is an emergent situation requiring release of the carpal tunnel and reduction and fixation of the wrist dislocation/fractures.

The American Academy of Orthopedic Surgeons has produced a clinical practice guideline for treating CTS.^5^ It includes 9 recommendations regarding treatment strategies based on the best level of evidence in the literature. The guideline recommends both nonsurgical and surgical treatments for early CTS. Nonsurgical treatment options include splinting, local steroid injection, ultrasound therapy, and oral steroids. Should nonsurgical treatment fail, the guideline recommends surgical carpal tunnel release.^2^

For the past 2 decades, the surgical options that physicians have used extensively to treat CTS have been both open carpal tunnel release and endoscopic carpal tunnel release surgery. The standard open technique provides superior exposure; the endoscopic technique results in a smaller scar. Both methods appear to be safe and effective and have high-level evidence to support their claims. The literature does not show any clear long-term differences in outcome measures between the 2 methods, and neither method appears to be clearly superior to the other method.^2^ Therefore, surgeons and patients should determine which procedure is most appropriate on a case-by-case basis. Other surgical techniques include minimal incision procedures, which require more blind dissection, and synovectomy, indicated only in cases of proliferative or invasive tenosynovitis. Postoperative splinting is optional and limited to 1 week or less to minimize debilitation.^1^

As with most surgeries, there are major complications of which it is necessary to be aware. The surgeon should avoid damage to the median nerve and its branches, damage to the ulnar nerve, and damage to the vascular structures in the palm of the hand. In addition, persistent weakness, pillar pain, and scar tenderness frequently occur after open carpal tunnel release.^6^

Last, because physicians often observe ulnar nerve compressive neuropathy along with CTS, one may consider releasing the ulnar nerve during carpal tunnel release surgery. However, the recommendation is not to release the ulnar nerve from Guyon's canal, as it is isolated from the CTS.^1^ Releasing the carpal tunnel typically relieves the pressure on the ulnar nerve as well.^7^ However, should the patient exhibit signs and symptoms of ulnar nerve compression, physicians should then consider releasing the ulnar nerve from Guyon's canal.
